# Host genetics and geography influence microbiome composition in the sponge *Ircinia campana*

**DOI:** 10.1111/1365-2656.13065

**Published:** 2019-09-03

**Authors:** Sarah M. Griffiths, Rachael E. Antwis, Luca Lenzi, Anita Lucaci, Donald C. Behringer, Mark J. Butler, Richard F. Preziosi

**Affiliations:** ^1^ Ecology and Environment Research Centre Manchester Metropolitan University Manchester UK; ^2^ School of Environment and Life Sciences University of Salford Salford UK; ^3^ Centre for Genomic Research, Institute of Integrative Biology University of Liverpool Liverpool UK; ^4^ Fisheries and Aquatic Sciences University of Florida Gainesville FL USA; ^5^ Emerging Pathogens Institute University of Florida Gainesville FL USA; ^6^ Department of Biological Sciences Old Dominion University Norfolk VA USA

**Keywords:** bacteria, core microbiome, eco‐evolutionary dynamics, genetic diversity, host–microbe interactions, microsatellites, Porifera

## Abstract

1. Marine sponges are hosts to large, diverse communities of microorganisms. These microbiomes are distinct among sponge species and from seawater bacterial communities, indicating a key role of host identity in shaping its resident microbial community. However, the factors governing intraspecific microbiome variability are underexplored and may shed light on the evolutionary and ecological relationships between host and microbiome.
2. Here, we examined the influence of genetic variation and geographic location on the composition of the *Ircinia campana* microbiome.
3. We developed new microsatellite markers to genotype *I. campana* from two locations in the Florida Keys, USA, and characterized their microbiomes using V4 16S rRNA amplicon sequencing.
4. We show that microbial community composition and diversity is influenced by host genotype, with more genetically similar sponges hosting more similar microbial communities. We also found that although *I. campana* was not genetically differentiated between sites, microbiome composition differed by location.
5. Our results demonstrate that both host genetics and geography influence the composition of the sponge microbiome. Host genotypic influence on microbiome composition may be due to stable vertical transmission of the microbial community from parent to offspring, making microbiomes more similar by descent. Alternatively, sponge genotypic variation may reflect variation in functional traits that influence the acquisition of environmental microbes. This study reveals drivers of microbiome variation within and among locations, and shows the importance of intraspecific variability in mediating eco‐evolutionary dynamics of host‐associated microbiomes.

## Graphical abstract

For the first time, the authors show that intraspecific genetic variation affects microbiome composition in a marine sponge (*Ircinia campana*), with positive correlations observed between genetic and microbiome similarity. This has significant implications for our understanding of the ecological and evolutionary relationships between host and microbiome in this important model system.

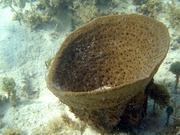

## 1 INTRODUCTION

A recent paradigm shift in biology has been the discovery of the breadth, diversity and importance of microbial communities associated with multicellular animals and plants. Termed the ‘microbiome’, these communities influence a number of traits associated with host health, physiology and development (Blaser, Bork, Fraser, Knight, & Wang, 2013; Gilbert, Jansson, & Knight, 2014), and as such have been the focus of attention in fields as diverse as human medicine and wildlife conservation (Kashyap, Chia, Nelson, Segal, & Elinav, 2017; Trevelline, Fontaine, Hartup, & Kohl, 2019). Host–microbiome systems are complex ecological communities encompassing an array of host–microbe and microbe–microbe interactions (Bauer, Kainz, Carmona‐Gutierrez, & Madeo, 2018; Coyte, Schluter, & Foster, 2015). Understanding the ecological and evolutionary nature of the relationship between hosts and their microbiome requires an understanding of the forces structuring these microbial communities, driven by both host and environment (Antwis et al., 2017).

Sponges (phylum Porifera) are considered valuable model systems in host–microbiome research due to the abundance and diversity within their associated microbial communities (Pita, Fraune, & Hentschel, 2016), with a total of 52 bacterial phyla and candidate phyla discovered among sponge hosts (Thomas et al., 2016). Sponge‐microbiome interactions are numerous and complex, and microbial symbionts may confer a number of benefits to their host including nutrition and waste metabolism (Freeman, Thacker, Baker, & Fogel, 2013; Karimi et al., 2018; Moitinho‐Silva et al., 2017; Thomas, Rusch, et al., 2010), acclimation to ocean acidification (Ribes et al., 2016), reduction in host surface fouling (On, Lau, & Qian, 2006) and production of compounds that deter predation of the sponge host (Garate, Blanquer, & Uriz, 2015). Sponge‐associated microbes are also of significant biotechnological interest due to their potential for production of novel, pharmaceutically active secondary metabolites (Thomas et al., 2010).

Sponge microbiome composition is predominantly host‐driven, with only a small degree of overlap with seawater microbial communities (Hentschel et al., 2002; Schmitt et al., 2012). Similarly, relatively few microbial taxa are shared across the phylum, and host species is a key determinant of microbiome composition (Blanquer, Uriz, & Galand, 2013; Pita, Turon, López‐Legentil, & Erwin, 2013; Schmitt et al., 2012; Thomas et al., 2016; Turon, Cáliz, Garate, Casamayor, & Uriz, 2018). This strong association is thought to be driven by a combination of vertical transmission of microbial associates (i.e., parent to offspring transmission) and horizontal transmission of seawater microbes to highly selective host environments (Fieth, Gauthier, Bayes, Green, & Degnan, 2016; Thacker & Freeman, 2012; Turon et al., 2018).

Despite the strong effect of host species identity, significant variation in microbiome composition is still present within host sponge species (Thomas et al., 2016; Turon et al., 2018). Intraspecific microbiome variation has been associated with environmental variation, such as geographic location (Fiore, Jarett, & Lesser, 2013; Luter et al., 2015; Swierts, Cleary, & de Voogd, 2018), depth (Morrow, Fiore, & Lesser, 2016), habitat (Cleary et al., 2013; Weigel & Erwin, 2017) and water quality (Luter et al., 2015). However, given the strength of host identity in structuring the microbiome, genetic variation within the species may also be significant. Indeed, host genotype influences microbiome composition in several systems, including plants (Wagner et al., 2016), fish (Uren Webster, Consuegra, Hitchings, & Garcia de Leaniz, 2018), amphibians (Griffiths et al., 2018), birds (Pearce, Hoover, Jennings, Nevitt, & Docherty, 2017) and mammals (Benson et al., 2010; Goodrich et al., 2014). However, host genotype and microbial variation have not yet been linked in sponges.

Noyer and Becerro (2012) found no significant relationship between host microsatellite diversity and bacterial communities analysed using denaturing gradient gel electrophoresis (DGGE) in *Spongia lamella*. However, DGGE has a lower resolution than current sequencing techniques, giving less information on community composition at lower taxonomic ranks. In a later study, Marino, Pawlik, López‐Legentil, and Erwin (2017) assessed correlations between latitude, sponge mitochondrial haplotype and microbiome composition in *Ircinia campana* from Florida and the Caribbean. However, cytochrome oxidase I haplotype correlated with location as well as microbiome composition, preventing the two variables from being disentangled. The role of host sponge genotype in structuring microbial communities is therefore still to be determined. This study addresses this gap in the literature, using highly polymorphic microsatellite markers and 16S rRNA Illumina sequencing to characterize *I. campana* populations and their associated microbial communities in two locations in the Florida Keys, USA.

## 2 MATERIALS AND METHODS

### 2.1 Sample collection and DNA extraction

In July 2014, we sampled *I. campana* (Caribbean vase sponge) individuals at two shallow (<2 m) nearshore hard bottom sites in the Florida Keys (FL, USA) separated by approximately 70 km: Long Key (24.81437, −80.8307) and Kemp Channel (24.6768, −81.4757). We took samples in a single collection instance at each site to eliminate temporal variability, with 20 individuals sampled per site. We cut a piece of tissue from each individual and immediately preserved it in 99% ethanol upon surfacing. We then replaced the ethanol, firstly to act as a rinse, removing loosely attached seawater bacteria, and secondly to prevent dilution of the ethanol to aid DNA preservation. We stored samples at −80°C until processing. Prior to DNA extraction, we dissected the tissue under a stereomicroscope using aseptic technique to remove commensal macro‐organisms. We then extracted total DNA with the DNeasy^®^ Blood and Tissue Kit (Qiagen), and normalized it to 1 ng/μl.

### 2.2 Microsatellite development and host genotyping

We developed a suite of 10 tri‐ and tetra‐nucleotide polymorphic microsatellite markers for *I. campana* using the pipeline implemented in the Palfinder Galaxy service (Griffiths et al., 2016) (Table S1, see Supporting Information for full details of methods). One locus (Icam34) performed well in individuals sampled from other localities (Griffiths et al. in prep, data not shown); however, it did not amplify well in the study populations. We therefore excluded Icam34 from further analysis in this study, thus using a total of nine loci. To fluorescently label PCR products, we used a three‐primer PCR method, using a fluorescently labelled universal primer and tagging the 5′ end of the forward primer with the universal primer sequence, as described by Culley et al. (2013). We carried out multiplex PCR amplifications using the Type‐it^®^ Microsatellite Kit (Qiagen) using the following PCR thermal cycling conditions: 95°C initial denaturation for 5 min, 28 cycles of 95°C for 30 s, 60°C/63°C for 90 s and 72°C for 30 s, and a final extension at 60°C for 30 min. PCR products were sized using the DNA Analyzer 3730 (Thermo Fisher Scientific) at the DNA Sequencing Facility at the University of Manchester, using the GeneScan^TM^ LIZ^®^ 1200 size standard (Thermo Fisher Scientific). We scored alleles with genemapper 3.7 (Thermo Fisher Scientific) and binned alleles in msatallele 1.03 (Alberto, 2009) in rstudio  1.1.442 for r 3.3.3 (RStudio Team, 2016; R Core Team, 2017).

### 2.3 Microbiome characterization

#### 2.3.1 PCR, library preparation and sequencing

PCR, sequencing and operational taxonomic unit (OTU) assignment were carried out at the Centre for Genomic Research, University of Liverpool, UK. We carried out amplification of the V4 region of the 16S rRNA gene in a two‐stage nested PCR in 5 μl reaction volumes using primers described in Caporaso et al. (2011). We used the following thermal cycling conditions: 15 × 95°C for 20 s, 65°C for 15 s and 70°C for 30 s; 1 × 72°C for 5 min. We purified PCR products using AMPure SPRI beads (Beckman Coulter), before entering into a second stage of PCR (conditions as above, 20 cycles) to incorporate Illumina sequencing adapter sequences containing indexes (i5 and i7) for sample identification. Following PCR, we purified the samples again and quality checked the amplicon libraries using a Qubit (Invitrogen) and a Fragment Analyzer (Agilent). We pooled the final libraries in equimolar amounts and used a Pippin Prep (Sage Science) to carry out size selection of 300–600 bp. We assessed quantity and quality of the library pool using a Bioanalyzer (Agilent) and qPCR with the Illumina^®^ Library Quantification Kit (Kapa Biosystems) on a LightCycler^®^ (Roche). We then conducted paired‐end (2 × 250 bp) sequencing on the Illumina MiSeq, with fragmented PhiX bacteriophage genome added to increase sequence complexity.

#### 2.3.2 Quality filtering and pre‐processing

We used casava 1.8.2 (Illumina) to base call and de‐multiplex indexed reads, and cutadapt 1.2.1 (Martin, 2011) to remove Illumina adapter and PCR primer sequences. We trimmed low‐quality bases from the reads using sickle 1.200 (Joshi & Fass, 2011) (minimum window quality score 20) and removed reads under 10 bp in length. Sequencing errors were corrected using the error‐correct module in spades 3.1.0 (Bankevich et al., 2012). We aligned read pairs using usearch 8 (Edgar, 2010) with the 'fast‐mergepairs' command and selected merged sequences of between 200 and 600 bp. We used BLASTN (Altschul, Gish, Miller, Myers, & Lipman, 1990) to search for PhiX sequences (GenBank GI:9626372) in each sample; matching sequences (*E*‐value < 10^–5^) were then filtered out. Sequences containing Ns were discarded to remove low‐quality reads.

We clustered sequences into OTUs with 99% sequence similarity. Two different clustering algorithms were used for OTU picking; the first implemented in vsearch 1.1.3 (Edgar, 2010) using the function ‘‐cluster‐smalmem’ with 99% identity threshold and the second in Swarm (Mahé, Rognes, Quince, de Vargas, & Dunthorn, 2014). We removed clusters containing fewer than two sequences to reduce error and merged the results from both clustering steps to create a non‐redundant sequence set. We conducted chimera detection in vsearch using both a de novo approach and a reference‐based approach with the silva 119 database. The reference‐based step found 12% of the sequences to be chimeras, which were removed for subsequent analyses, while none were found using the de novo approach. We used the ‘usearch_global’ function in vsearch to define the abundance of each OTU and taxonomically classified these in qiime 1.9.0 (Caporaso et al., 2010) using pick_rep_set.py to select the most representative sequence in the OTU and assign_taxonomy.py to match sequences to those in the silva 119 database (Quast et al., 2013). We produced an OTU count table for all samples, and exported this and the taxonomic classification as a biom file. We imported this into rstudio using the phyloseq package (McMurdie & Holmes, 2013) for subsequent statistical analyses. We converted OTU count data to relative abundance for subsequent compositional and beta diversity analyses.

We also created a rarefied dataset for use in alpha diversity analyses, as sequencing depths among samples were uneven. Repeated subsampling (33 repetitions) was carried out on the OTU count table at sampling depths from 2,000 to 350,000 in QIIME (multi_rarefaction.py), following which we calculated Chao1 alpha diversity and plotted rarefaction curves (Figure S1). We then created a rarefied dataset (single_rarefaction.py) by repeatedly subsampling (without replacement) at a depth of 173,000 sequences; samples with fewer sequences were removed from subsequent analysis (leaving *n* = 12 for Kemp Channel and *n* = 13 for Long Key). We used this dataset for alpha diversity analyses as described below; we also repeated our beta diversity analyses using this dataset, which produced similar results to the non‐rarefied dataset (data not shown).

### 2.4 Statistical analyses

#### 2.4.1 Host genetics

We tested for linkage disequilibrium between microsatellite loci using genepop on the web 4.2 (Rousset, 2008), correcting significance values for multiple tests using Benjamini and Yekutieli's (2001) correction with the R function *p.adjust*. We estimated null allele frequencies in freena (Chapuis & Estoup, 2007) using the EM algorithm (Dempster, Laird, & Rubin, 1977). We calculated *F*_ST_ between the two sites and corrected for null allele presence using the ENA method as described in Chapuis and Estoup (2007) and implemented in freena.

We calculated pairwise Euclidean genetic distances between individuals from the multi‐locus genotypes using genodive 2.0b27 (Meirmans & Van Tiendener, 2004). In distance‐based calculations, null alleles and missing data can bias results, overestimating differences between samples (Chapuis & Estoup, 2007). Thus, we first filled in missing data based on overall allele frequencies (11.8% in Long Key and 7.6% in Kemp Channel). We then used these distances to conduct a principal coordinates analysis (PCoA) in genalex 6.503 (Peakall & Smouse, 2012).

#### 2.4.2 Microbiome composition

We conducted analyses in rstudio using the phyloseq (McMurdie & Holmes, 2013), vegan (Oksanen et al., 2018) and microbiome (Lahti 2017) packages. We used a PERMANOVA (adonis) to test for significant differences in microbiome composition between sites using Bray–Curtis dissimilarities. We calculated the core microbiome of individual samples using a detection threshold of 0.001% and a prevalence threshold of 100% (i.e., a given OTU must be present in all individuals, with a relative abundance of at least 0.001%). We identified the core OTUs and then calculated the proportion of the total microbiome that these represented. We then repeated the core microbiome analysis with the data agglomerated to genus level.

#### 2.4.3 Host genotype–microbiome analyses

We produced pairwise microbial community distance matrices between individuals across both sites, and for each site individually, using distance matrices based on Chao1 values as a measure of alpha diversity, and Jensen–Shannon divergence (JSD) and Bray–Curtis dissimilarity as measures of beta diversity. We tested for correlations between each microbial distance matrix (Chao1, JSD and Bray–Curtis) and host genetic distance matrix (Euclidean) using Mantel tests with 999 permutations. We repeated the analyses with a further, more conservative genetic data file, removing loci with high null allele frequencies and high proportions of missing data. Following removal of loci with high (>0.16) null allele frequencies (Icam24, Icam26 and Icam10 in both sites, Icam3 in Kemp Channel samples), missing data were only present in Icam23 (0.077) and Icam3 (0.462) in Long Key. We then removed Icam3 at Long Key, giving final datasets of five loci for each site.

We also extracted Bray–Curtis distance matrices for core microbiota across and within sites at both the OTU and genus levels, and used Mantel tests to test for correlation with the full and reduced genetic distance matrices. As the taxonomic composition of the core does not vary across individuals, this metric describes variation in relative abundances of these core taxa.

## 3 RESULTS

### 3.1 Population genetics

We found no identical multi‐locus genotypes in the dataset, indicating no clones were present among the sampled individuals. Across both sites, all loci were polymorphic, ranging up to 18 alleles per locus, but two of the loci were monomorphic in Kemp Channel (Icam32 and Icam4) (Table S2). No significant linkage disequilibrium occurred between any pairs of loci. Null allele frequencies and the proportion of missing alleles (genotyping failures) were high for many loci, and heterozygosity deficiencies were observed in many cases (Table S2).

**Figure 1:**
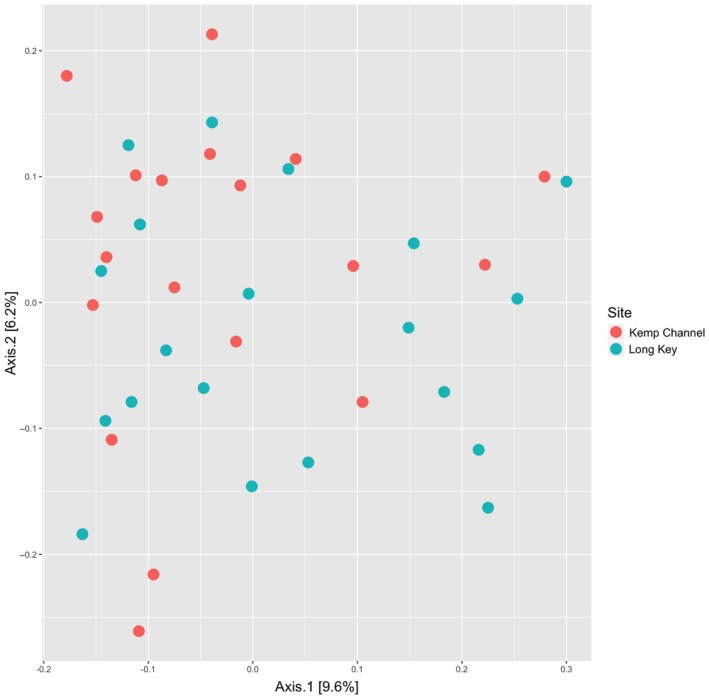
Principal coordinates analysis (PCoA) showing Euclidean genetic distances among *Ircinia campana* individuals at Long Key and Kemp Channel

Pairwise genetic differentiation between Long Key and Kemp Channel was low (*F*_ST_ = 0.021). The first and second principle coordinates of the PCoA explained only 15.81% of the total variation among the samples, and the individuals are not separated by site (Figure 1). The sites can therefore be considered to be well‐mixed genetically.

### 3.2 Microbial community composition

We successfully conducted PCR amplification and Illumina sequencing on 34 samples (17 each from Long Key and Kemp Channel). Between 97.10% and 99.61% of reads were assembled per sample. One sample from Kemp Channel yielded far fewer assembled sequences than the remaining samples (28,163) and was removed from the analyses, leaving samples with between 118,370 and 426,014 assembled sequences (mean = 241,448 ± 13,758 *SE*, Figure S1). Between 80.77% and 87.57% of the filtered sequence set could be aligned to a taxon, with 31,567 OTUs found across all samples, and individual sponges ranging from 4,165 to 14,503 OTUs (Figure S1). Among all OTUs, we detected a total of 22 bacterial phyla and one archaeal phylum. The most abundant phylum was Chloroflexi (62.6% of the total reads), followed by Proteobacteria (17.5%), Acidobacteria (6.4%), PAUC34f (4.3%), SBR1093 (3.8%), Gemmatimonadetes (1.6%) and Actinobacteria (1.5%) (Figure S2). The remaining phyla formed less than 1% of the total reads. Within Chloroflexi, Anaerolineae was the most dominant class, forming large proportions of the microbiomes of all samples (Figure S3).

**Figure 2:**
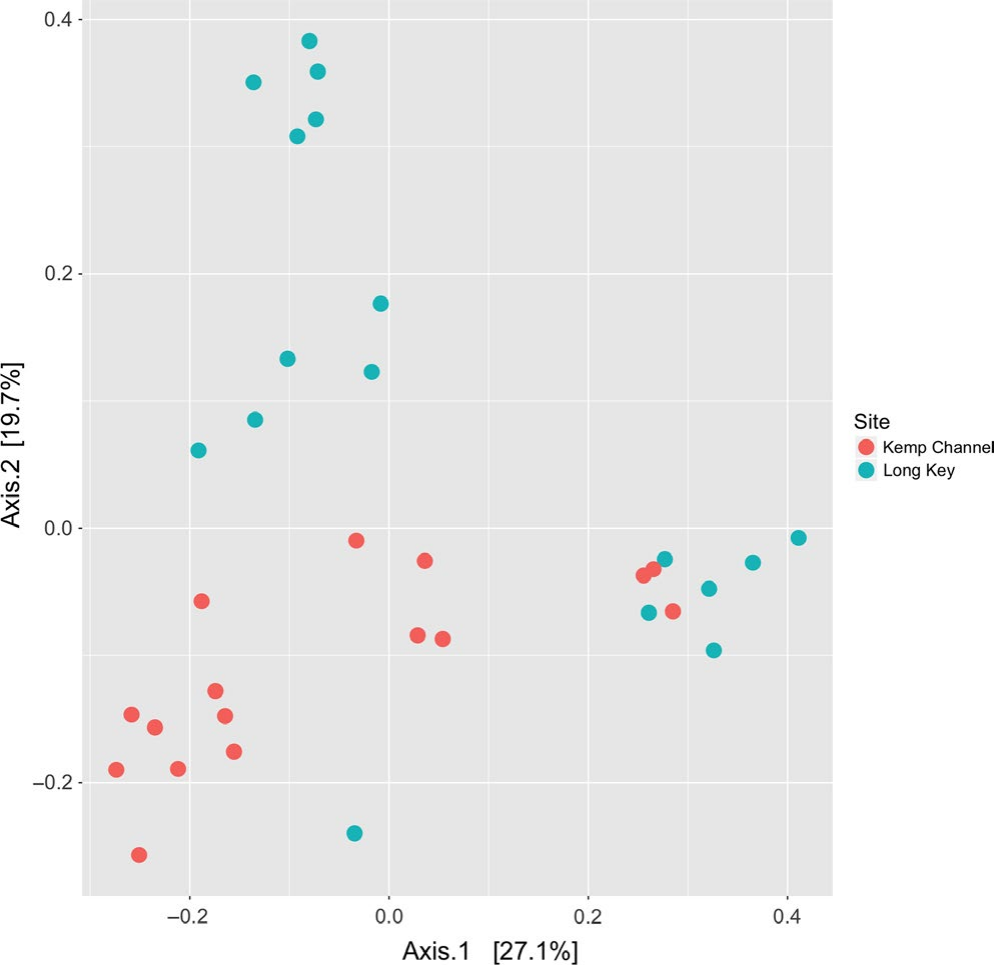
Principle coordinates analysis (PCoA) showing Bray–Curtis distances among microbiomes of *Ircinia campana* from Long Key and Kemp Channel

There was a statistically significant difference in microbiome composition between sponges at Long Key and Kemp Channel (adonis, *F*_1,31_ = 4.391, *R* ^2^ = .124, *p* = .001; Figure 2), with 12.4% of the variation in microbiome composition explained by site. These compositional differences are evident, albeit subtle, at both the phylum and class levels (Figures S2 and S3).

At the genus level, the core microbiome comprised 69.6% (±1.7 *SE*) of the total reads for sponges in Long Key and 63.0% (±1.6) for sponges in Kemp Channel. These genera included *Desulfovibrionales*,* Chloroflexi*,* Pseudospirillium*,* PAWS52f*,* Nitrosococcus*,* Rhodovulum*,* Defluviicoccus*,* Acidobacteria*,* OM75 clade*,* Granulosicoccus*,* Nitrospira*,* Cerasicoccus*,* Actinobacterium MSI70*,* Candidatus Nirtosopumilus*,* Acidobacterium*,* PAUC32f*,* Truepera*,* PAUC43f*,* Synechococcus* and a number of unidentified Proteobacteria genera. Out of a total of 31,567 OTUs, we only identified two in the core microbiome; one assigned to Proteobacteria and the other an unidentified Bacteria. Together these comprised 1.2% (±0.2) of the total microbiome for sponges in Long Key and 1.3% (±0.1) in Kemp Channel, and had the third (Proteobacteria sp.) and eleventh (Bacteria sp.) highest relative abundances of all OTUs in the total microbiome.

### 3.3 Relationship between host genotype and microbiome composition

**Figure 3:**
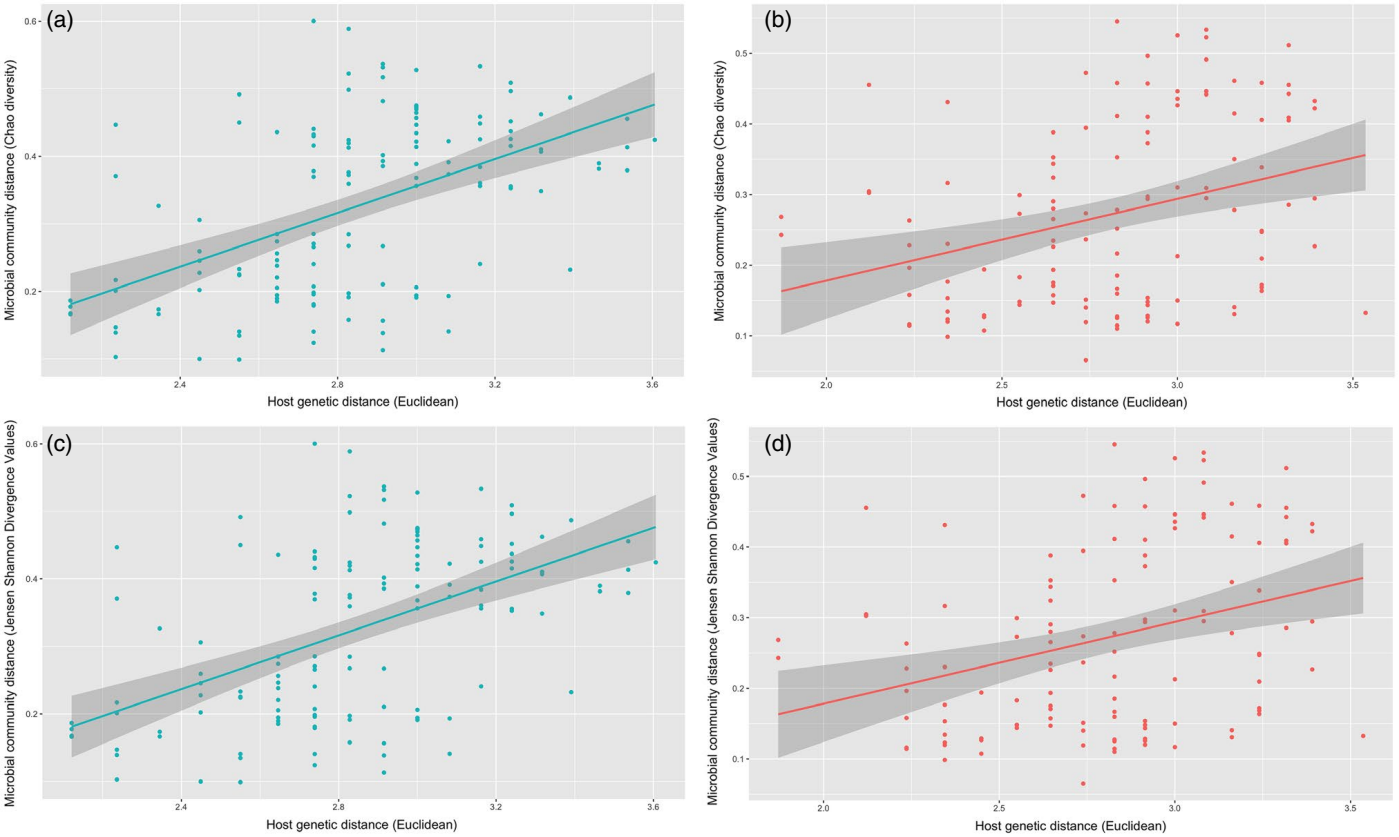
Scatter plot with regression lines showing correlations between pairwise host Euclidean genetic distances (using nine microsatellite genotypes) and microbial community Chao1 distances (alpha diversity) in *Ircinia campana* at (a) Long Key, and (b) Kemp Channel, and host genetic Euclidean distances and microbial community Jensen–Shannon divergence values (beta diversity) at (c) Long Key, and (d) Kemp Channel. Shaded areas show 95% confidence intervals

We found statistically significant, positive relationships between genetic distance and microbial community dissimilarity when considering both alpha diversity (Chao1) and beta diversity (Bray–Curtis and JSD distances) and when using the full and reduced microsatellite datasets (Table 1; Figure 3). However, there were no significant relationships between genetic distance and core microbiome distance at either the OTU or genus level when using either of the genetic datasets (all *p* > .100).

**Table 1:** Results of Mantel tests between genetic distance matrices and microbiome distance matrices of *Ircinia campana*. Genetic distances were based on either 10 (‘full genetic dataset’) or five (‘reduced genetic dataset’) microsatellites

Location	Microbiome dissimilarity metric	Genetic dataset	*r* value	*p* value
Across sites	Chao1	Full	.410	.001
		Reduced	.347	.001
	Bray–Curtis	Full	.211	.002
		Reduced	.139	.025
	JSD	Full	.278	.001
		Reduced	.206	.003
Long Key	Chao1	Full	.503	.001
		Reduced	.264	.005
	Bray–Curtis	Full	.465	.001
		Reduced	.232	.006
	JSD	Full	.483	.001
		Reduced	.236	.009
Kemp Channel	Chao1	Full	.297	.033
		Reduced	.340	.023
	Bray–Curtis	Full	.280	.013
		Reduced	.228	.039
	JSD	Full	.307	.014
		Reduced	.277	.033

## 4 DISCUSSION

Host genotype had a significant effect on microbiome diversity and composition in *I. campana*, both across and within sites. More genetically‐similar sponges hosted more similar microbial communities, in terms of both richness and composition. Between sites, sponge microbiomes significantly differed in composition, despite no genetic differentiation between the sponge populations. These results indicate that both environment and host genetics influence intraspecific microbiome variability in *I. campana* and that these drivers vary in influence by spatial scale.

### 4.1 Host genotype

Host genetic similarity and microbiome similarity were positively correlated, both in terms of microbiome alpha‐ and beta diversity. This relationship may be driven by vertical transmission of microbial communities, with sponges that are more genetically similar by descent hosting more similar microbiomes. Evidence for vertical transmission of the microbiome has been observed in the sympatric congener *Ircinia felix* (Schmitt, Weisz, Lindquist, & Hentschel, 2007), as well as other sponge species (Ereskovsky, Gonobobleva, & Vishnyakov, 2005; Lee, Chui, Wong, Pawlik, & Qian, 2009; Sharp, Eam, Faulkner, & Haygood, 2007; Sipkema et al., 2015), and is thought to be a significant driver of the high host species fidelity of microbiomes in sponges. Many evolutionary advantages can be gained from the inheritance of parental microbiomes, as favourable symbionts that are important for host health and physiology are already present in growing larvae.

Horizontal transmission of microbes from the environment also contributes to the sponge microbiome (Fieth et al., 2016; Maldonado & Riesgo, 2009; Sipkema et al., 2015; Turon et al., 2018). As such, ecological interactions with seawater microbes could be key in shaping the microbiome. The relationships observed in this study may therefore be driven by host genotype‐specific selection of seawater microbes.

Selection of environmental microbes imposed by host genetic variation could result from secondary metabolites produced by the sponge, which are highly diverse (Genta‐Jouve & Thomas, 2012), and include antimicrobial compounds (Kelman et al., 2001). Using an experimental approach, Tout et al. (2017) showed that seawater bacteria exhibit chemotaxis to cellular extracts isolated from a sponge, with particular enrichment of bacterial taxa that are commonly found in sponges. As sponge secondary metabolites can be intraspecifically variable (Noyer, Thomas, & Becerro, 2011; Puyana et al., 2015), the production of genotype‐specific compounds could attract varying seawater microbes to the sponge microbiome.

Alternatively, genetic variation may encode variable responses in the host immune system to microbes in the environment. In other species, polymorphism in immunity‐related genes has been found to affect microbiome composition (Bolnick et al., 2014; Kubinak et al., 2015; Pearce et al., 2017) and responses to pathogenic bacteria (Lazzaro, Sceurman, & Clark, 2004). In addition, genotype‐specific immune response, and varying gene expression patterns in response to a potentially pathogenic bacteria, have been observed in the coral *Acropora millepora* (Wright et al., 2017). Although they do not have an acquired immune system, sponges have a relatively sophisticated innate immune system (Müller & Müller, 2003), which has been speculated to aid the maintenance of distinct extracellular microbial communities in the mesohyl tissue where phagocytosis of food bacteria takes place (Wehrl, Steinert, & Hentschel, 2007; Wilkinson, Garrone, & Vacelet, 1984). This system includes receptor proteins at the interface between the organism and the environment that can recognize and differentiate bacteria (Wiens et al., 2005, 2007). There is currently no evidence that immune response varies intraspecifically in sponges. However, there is evidence of polymorphism of the *Amphimedon queenslandica AqNLR* (nucleotide‐binding domain and Leucine‐rich repeat containing) genes, which are pattern recognition receptors involved in detecting and binding a range of microbial ligands (Degnan, 2015).

Further to these potential mechanisms, the sponge itself cannot be considered in isolation; selection of seawater bacteria is likely to be performed by the entire holobiont. The timing and order in which microbes join a sponge microbiome may have secondary effects on determining succession and ultimately community composition (historical contingency; Costello, Stagaman, Dethlefsen, Bohannan, & Relman, 2012), with competitive interactions occurring among community members (Esteves, Cullen, & Thomas, 2017). Because of this, influence of the host genotype on even a relatively small proportion of the microbiome could increase its reach in shaping community composition.

### 4.2 Geographic location

Location was the largest driver of microbiome structure in this study. Although the sampling sites are only approximately 70 km apart, location accounted for 12.4% of the total microbiome variation observed across samples. Microbiomes vary within species by geographic location in a number of benthic marine organisms (Pantos, Bongaerts, Dennis, Tyson, & Hoegh‐Guldberg, 2015; Rubio‐Portillo, Kersting, Linares, Ramos‐Esplá, & Antón, 2018; van de Water, Allemand, & Ferrier‐Pagès, 2018), including in some sponge species (Fiore et al., 2013; Luter et al., 2015; Marino et al., 2017; Swierts et al., 2018), although this finding is not universal (Pita, López‐Legentil, & Erwin, 2013; Pita, Turon, et al., 2013). Marino et al. (2017) showed a latitudinal gradient in microbiome composition in *I. campana* in the Caribbean, which also correlated with host mitochondrial haplotype. Our results show that even on a relatively local spatial scale (i.e., within the Florida Keys), microbiomes of *I. campana* can vary among sampling sites.

In this study, we did not sample seawater bacterial communities or collect environmental data, as investigating environmental effects was not the objective of this study. Instead, we sampled two sites as a form of replication to investigate host genetics. However, because there was effectively no genetic differentiation between the sponge populations at each site, the microbiome differences found between sites indicate that environmental variation drives *I. campana* microbiome composition at larger spatial scales. The environmental parameters responsible for this pattern remain unknown. However, host genotype also had a significant effect on microbiome composition when considered across locations. In addition, there appears to be between‐site variation in the strength of host genotype–microbiome relationships. Mantel test statistic values were mostly higher for Long Key than those for Kemp Channel; this could be the result of genotype x environment interactions and could extend the influence of genotype at larger spatial scales.

### 4.3 Core microbiome

Despite a significant effect of host genotype on total microbiome composition, we did not find any effect of host genotype on core microbiome composition at the genus or OTU level. As the core was defined in this study as taxa found in all individuals, composition refers here to variation in relative abundance of the same microbial phylotypes among individuals. Therefore, our results show that while genotype exerts an effect on non‐core microbiome taxa, it does not drive abundances of core taxa. This lends support to the theory that horizontal transmission has an important role in forming the core microbiome (Turon et al., 2018), as we may expect vertical transmission to produce significant genotype–core microbiome relationships. However, some refinement of the core microbiome concept and, in particular, the associated methodologies to define and identify ‘core’ taxa may be required to further our understanding of their significance, role and transmission.

At the 99% OTU level, the core microbiome in our study was comprised of two OTUs, which made up 1.2% and 1.3% of the total microbiome in Long Key and Kemp Channel, respectively. This core community appears small compared with results reported in some previous studies on sponges (Marino et al., 2017; Turon et al., 2018). Marino et al. (2017) found 119 core OTUs among 18 *I. campana* individuals, comprising 79.2%–87.0% of the total microbiome. However, Marino et al. used 97% OTU clustering, which means individual OTUs are likely to encompass wider microbial taxonomic variation in contrast to 99% clustering of OTUs as used in this study. The core microbial genera in our study formed 63% and 69% of the total microbiome in Long Key and Kemp Channel, respectively, suggesting that the differences observed relative to previous work primarily reflect methodical differences (Astudillo‐García et al., 2017). Furthermore, the higher the number of replicates, the smaller the apparent ‘core’ microbiome appears (Turon et al., 2018), and in this study, the number of within‐species replicates we used (*n* = 33) was substantially larger than in many previous studies.

The core microbiome concept aims to identity stable, functionally important members of the microbiome, rather than transient or opportunistic members (Hernandez‐Agreda, Gates, & Ainsworth, 2017; Shade & Handelsman, 2012). *Ircinia campana* appears to have a strong, possibly symbiotic, relationship with the two core OTUs observed, indicating a potentially important role in holobiont function. However, the larger genus‐level core observed may be due to a level of functional redundancy within microbial genera, with characteristics at higher taxonomic ranks being more important for successful transmission and stability than OTU‐level characteristics. As such, defining a core in terms of wider phylogenetic or functional groups may therefore be more useful than a strict OTU/ species‐level approach (Turnbaugh et al., 2009). Furthermore, as our findings show that microbiomes vary by genotype, identifying stable associations using the host species‐level core microbiome approach may obscure genotype‐specific host–microbe symbioses.

### 4.4 Concluding remarks

We show that genetic diversity has an important influence in shaping microbiome composition in *I. campana*. These results highlight the potential for intraspecific genetic diversity to impact ecological dynamics within sponge–microbe relationships and demonstrate an eco‐evolutionary relationship between sponges and microbial communities. Further work on the mechanisms underlying host genotype–microbiome relationships will aid our understanding of the nature of sponge‐microbiome associations. Furthermore, understanding drivers of interspecific microbiome variability is important in the context of global climate change. Ocean warming and acidification are predicted to change microbial communities both within the environment and in host‐associated microbiomes, with huge implications for health and survival of marine species and their ecosystems (Lesser, Fiore, Slattery, & Zaneveld, 2016; Qiu et al., 2019). For example, ocean warming in the Mediterranean triggered microbial imbalances in *I. fasciculata*, which have been implicated in disease and mass mortalities (Blanquer, Uriz, Cebrian, & Galand, 2016). In Florida Bay, *I. campana* populations have already suffered numerous mass mortality events (Butler et al., 1995; Stevely et al., 2010) due to cyanobacterial blooms caused by decades of ecosystem instability (Butler & Dolan, 2017; Butler et al., 2018; Fourqurean & Robblee, 1999; Kearney et al., 2015; Robblee et al., 1991). Microbiomes are potentially important in acclimation and resilience to climate change scenarios in marine organisms (Ribes et al., 2016; Webster & Reusch, 2017). With this in mind, understanding individual‐level drivers of microbiome variation may assist in species management and conservation in the face of future stressors.

## AUTHORS' CONTRIBUTIONS

S.M.G. and R.F.P. conceived and designed the study; S.M.G., R.F.P., M.J.B. and D.C.B. secured funding; S.M.G., M.J.B. and D.C.B. collected the samples; S.M.G. and A.L. carried out the laboratory work; S.M.G., R.E.A. and L.L. conducted the analysis; S.M.G. and R.E.A. wrote the manuscript; all authors revised the manuscript.

## Data Availability

16S rRNA amplicon sequence data are available from the NCBI SRA (accession number PRJNA506340). Microsatellite loci sequences are logged in NCBI GenBank under accession numbers MF987878 to MF987887. Raw Illumina sequencing data from *Ircinia campana* used for microsatellite development is deposited in the NCBI SRA (accession number PRJNA528609). The code used for analyses in R is available as a RMarkdown file in the Supporting Information.
