# Supplementary material for: Host genetics and geography influence microbiome composition in the sponge Ircinia campana
Source: J Anim Ecol. 2019 Sep 3;88(11):1684–95. doi: 10.1111/1365-2656.13065 (PMC6899969; doi:10.1111/1365-2656.13065)
Supplement: Supplementary file 1 [file JANE-88-1684-s001.docx]

**Griffiths et al. Supporting Information**

**Host genetics and geography influence microbiome composition in the sponge *Ircinia campana***

**Methods**

*Developing microsatellite markers for Ircinia campana*

A single *Ircinia campana* individual was used for microsatellite development. To avoid contamination from microbial DNA and associated invertebrates, we dissected the sponge tissue under a stereomicroscope, and separated prokaryote and eukaryote cells (as far as possible) using the centrifugation method of Freeman & Thacker (2011). We extracted DNA from the ‘eukaryotic’ cell pellet with the DNeasy® Blood and Tissue Kit (Qiagen), and used 50ng of DNA for library construction with the Nextera® DNA Sample Preparation Kit. Paired-end sequencing on the Illumina MiSeq (2 x 250 base pairs (bps)) resulted in 2 x 1,939,933 reads. Library preparation and sequencing was carried out at the University of Manchester’s Genomic Technologies Facility.

We used the Palfinder Galaxy Service (Griffiths et al. 2016), a customised Galaxy instance (Afgan *et al.* 2018) for microsatellite development, to process the raw data, locate microsatellite regions and design suitable primers. We quality filtered and trimmed reads with the Trimmomatic 0.32 tool (Bolger, Lohse & Usadel, 2014), using the ‘sliding window’ feature with a window size of 4 bps and a quality threshold of Phred score 20. Low quality reads at the start and end of reads were trimmed using the ‘leading’ and ‘trailing’ functions, both set to a value of 3. Following quality filtering, 2 x 1,175,084 reads remained. We then used the pal_finder 0.02.04 tool (Castoe, Poole & Koning, 2012) to locate microsatellite regions with at least eight repeated units. Where possible, Primer3 2.0.0 (Koressaar & Remm 2007; Untergasser *et al.* 2012) was used to design primers.

We selected 36 tri- and tetra-nucleotide loci for testing; these were shown to contain perfectly repeating motifs, and had primer sequences that were only found once in the entire set of reads. We carried out PCR amplifications with the Type-it® Microsatellite Kit (Qiagen) in 5μl reaction volumes with the following thermal cycling conditions: 95°C initial denaturation for 5 minutes, 28 cycles of 95°C for 30 seconds, 60°C for 90 seconds and 72°C for 30 seconds, and a final extension at 60°C for 30 minutes. After this process, eighteen loci produced clear bands on agarose gels. To further examine their suitability and size ranges, we repeated the PCRs as above, but with a universal primer tail fluorophore labelling system using the M13(-21) tail (TGTAAAACGACGGCCAGT) and the 6-FAM dye to fluorescently label PCR products (Culley *et al.* 2013). These were then analysed on a DNA Analyzer 3730 (Thermo Fisher Scientific) at the University of Manchester DNA Sequencing Facility with the GeneScan^TM^ LIZ® 1200 (Thermo Fisher Scientific) size standard. We then viewed the electropherograms in Genemapper v3.7 (Thermo Fisher Scientific); 10 loci were suitable for further use as they exhibited easy-to-score peaks, consistent amplification and minimal stutter. We designed two multiplexes incorporating the 10 loci using Multiplex Manager (Holleley & Geerts 2009), with the following universal primers to fluorescently label PCR products: 6FAM- TGTAAAACGACGGCCAGT (M13-21; Culley et al., 2013), HEX- CGGAGAGCCGAGAGGTG (Tail D; Blacket et al., 2012) and PET-CACTGCTTAGAGCGATGC (M13 modified B; Culley et al., 2013) (Table S1). We carried out multiplex PCR amplifications out using the Type-it® Microsatellite Kit (Qiagen) in 5μl reaction volumes with 2μM of the forward primer, 2μM of the reverse primer, and 0.5μM of the third universal primer, using the following PCR thermal cycling conditions: 95°C initial denaturation for 5 minutes, 28 cycles of 95°C for 30 seconds, 60°C (multiplex A) or 63°C (multiplex B) for 90 seconds and 72°C for 30 seconds, and a final extension at 60°C for 30 minutes.

**References**
1.Afgan, E., Baker, D., Batut, B., van den Beek, M., Bouvier, D., Čech, M., *et al.* (2018). The Galaxy platform for accessible, reproducible and collaborative biomedical analyses: 2018 update. *Nucleic Acids Res.*, 46, W537–W544

2.Blacket, M., Robin, C., Good, R., Lee, S. & Miller, A. (2012). Universal primers for fluorescent labelling of PCR fragments- an efficient and cost-effective approach to genotyping by fluorescence. *Mol. Ecol. Resour.*, 12, 456–63

3.Bolger, A.M., Lohse, M. & Usadel, B. (2014). Trimmomatic: a flexible trimmer for Illumina sequence data. *Bioinformatics*, 30, 2114–2120

4.Castoe, T., Poole, A., de Koning, A., Jones, K., Tomback, D., Oyler-McCance, S., *et al.* (2012). Rapid microsatellite identification from Illumina paired-end genomic sequencing in two birds and a snake. *PLoS One*, 7, e30953

5.Culley, T.M., Stamper, T.I., Stokes, R.L., Brzyski, J.R., Hardiman, N. a., Klooster, M.R., *et al.* (2013). An efficient technique for primer development and application that integrates fluorescent labeling and multiplex PCR. *Appl. Plant Sci.*, 1, 1300027

6.Freeman, C.J. & Thacker, R.W. (2011). Complex interactions between marine sponges and their symbiotic microbial communities. *Limnol. Oceanogr.*, 56, 1577–1586

7.Griffiths, S.M., Fox, G., Briggs, P.J., Donaldson, I.J., Hood, S., Richardson, P., *et al.* (2016). A Galaxy-based bioinformatics pipeline for optimised, streamlined microsatellite development from Illumina next-generation sequencing data. *Conserv. Genet. Resour.*, 8, 481–486

8.Holleley, C.E. & Geerts, P.G. (2009). Multiplex Manager 1.0: a cross-platform computer program that plans and optimizes multiplex PCR. *Biotechniques*, 46, 511–7

9.Koressaar, T. & Remm, M. (2007). Enhancements and modifications of primer design program Primer3. *Bioinformatics*, 23, 1289–91

10.Untergasser, A., Cutcutache, I., Koressaar, T., Ye, J., Faircloth, B.C., Remm, M., *et al.* (2012). Primer3- new capabilities and interfaces. *Nucleic Acids Res.*, 40, e115

**Table S1**

Characterisation of 10 *Ircinia campana* microsatellite loci and two multiplexes.

| **Locus name** | **Motif** | **Primer sequences (5’🡪 3’)** | **MP** | **L** | **Na** | **Size range**  **(bp)** | **GenBank accession no.** |
| --- | --- | --- | --- | --- | --- | --- | --- |
| Icam10 | ATC | F: TATGCCGATACCCAATGACATCACC  R: GCTGTGTGGATACAGTAAATGTCCAACG | A | 3 | 19 | 331-414 | MF987878 |
| Icam3 | ATAC | F: ACAAGTGCAGCATGGAGAATGTGC  R: CCTGTGTGTATCCATCACAAGTGTCC | A | 1 | 32 | 503-616 | MF987882 |
| Icam4 | AATC | F: ACAGCATGGCAGTGTTTTCTGATCG  R: ACATATCGACAGGACAAGCTGATGG | A | 1 | 19 | 137-275 | MF987879 |
| Icam18 | ATAC | F: TCTTGGCAGCCTTAGATTGAACAGC  R: TGCAGTGGCTTCTATGACTTTAAACAAAGC | A | 2 | 96 | 346-617 | MF987883 |
| Icam31 | ATAC | F: TGTTATAAACTGCGGCTATGGATGTACG  R: GTCATGCATCCAGAATGACCACTCC | A | 1 | 21 | 343-394 | MF987880 |
| Icam32 | TTC | F: GCATTACAAATAGGTTGGCCTTTGTGG  R: GCAAGAAAGCAAATGTTAGAGCGAACC | A | 3 | 4 | 277-282 | MF987887 |
| Icam23 | ATAC | F: TGCTGGACAAGAGAGGTTTCACTGC  R: TTGAACTCAGGCCTCCTGACATACG | B | 1 | 97 | 396-825 | MF987884 |
| Icam24 | ATAC | F: CATTGGTTAACAAACCTATAGCAACCC  R: ACTGCCTGTACAAACATTAACATGC | B | 2 | 83 | 428-830 | MF987885 |
| Icam26 | ATAC | F: GTCTTGTGTGGACCTTCAGATCACC  R: GGGATGATGTGATAAAGCATTTCC | B | 1 | 21 | 350-409 | MF987886 |
| Icam34 | ATAC | F: TGCACATAACCCCTATTTTCTCATGCC  R: GCTATTTTCGTGCCATGATTTCAGC | B | 3 | 58 | 201-459 | MF987881 |

MP: multiplex; L: tail sequence/ florescent label combination (1: 6FAM- TGTAAAACGACGGCCAGT; 2: HEX- CGGAGAGCCGAGAGGTG;

3: PET- CACTGCTTAGAGCGATGC); Na: number of alleles per locus; bp: base pairs. Size range and number of alleles include data taken from a larger Caribbean-wide

dataset (Griffiths et al., unpublished).

**Table S2**

Summary statistics for *Ircinia campana* microsatellites at Long Key and Kemp Channel.

|  | **Icam23** | **Icam24** | **Icam26** | **Icam10** | **Icam18** | **Icam3** | **Icam31** | **Icam32** | **Icam4** | **Overall** |
| --- | --- | --- | --- | --- | --- | --- | --- | --- | --- | --- |
|  |  |  |  |  |  |  |  |  |  |  |
| **Long Key** |  |  |  |  |  |  |  |  |  |  |
|  |  |  |  |  |  |  |  |  |  |  |
| Number of alleles | 18 | 10 | 7 | 5 | 18 | 7 | 3 | 2 | 2 | 8.000 |
| Proportion of missing data | 0.118 | 0.294 | 0.059 | 0.176 | 0.000 | 0.412 | 0.000 | 0.000 | 0.000 | 0.118 |
| Null allele frequency | 0.138 | 0.426 | 0.299 | 0.404 | 0.147 | 0.000 | 0.083 | 0.000 | 0.000 | 0.166 |
| Observed heterozygosity | 0.667 | 0.083 | 0.250 | 0.000 | 0.647 | 0.900 | 0.471 | 0.235 | 0.059 | 0.368 |
| Expected heterozygosity | 0.971 | 0.962 | 0.798 | 0.725 | 0.961 | 0.800 | 0.561 | 0.213 | 0.059 | 0.672 |
|  |  |  |  |  |  |  |  |  |  |  |
|  |  |  |  |  |  |  |  |  |  |  |
| **Kemp Channel** |  |  |  |  |  |  |  |  |  |  |
|  |  |  |  |  |  |  |  |  |  |  |
| Number of alleles | 10 | 12 | 6 | 5 | 17 | 4 | 6 | 1 | 1 | 6.889 |
| Proportion of missing data | 0.000 | 0.250 | 0.000 | 0.125 | 0.000 | 0.312 | 0.000 | 0.000 | 0.000 | 0.076 |
| Null allele frequency | 0.174 | 0.294 | 0.294 | 0.287 | 0.033 | 0.244 | 0.187 | 0.001 | 0.001 | 0.168 |
| Observed heterozygosity | 0.375 | 0.333 | 0.250 | 0.214 | 0.875 | 0.091 | 0.375 | 0.000 | 0.000 | 0.279 |
| Expected heterozygosity | 0.754 | 0.955 | 0.775 | 0.731 | 0.950 | 0.418 | 0.704 | 0.000 | 0.000 | 0.587 |
|  |  |  |  |  |  |  |  |  |  |  |

**Figure S1**

Rarefaction curves showing mean observed alpha diversity for microbial 99% OTUs at increasing sequencing depths in each *Ircinia campana* sample. Solid lines represent samples from Long Key, and dashed lines represent samples from Kemp Channel.

**
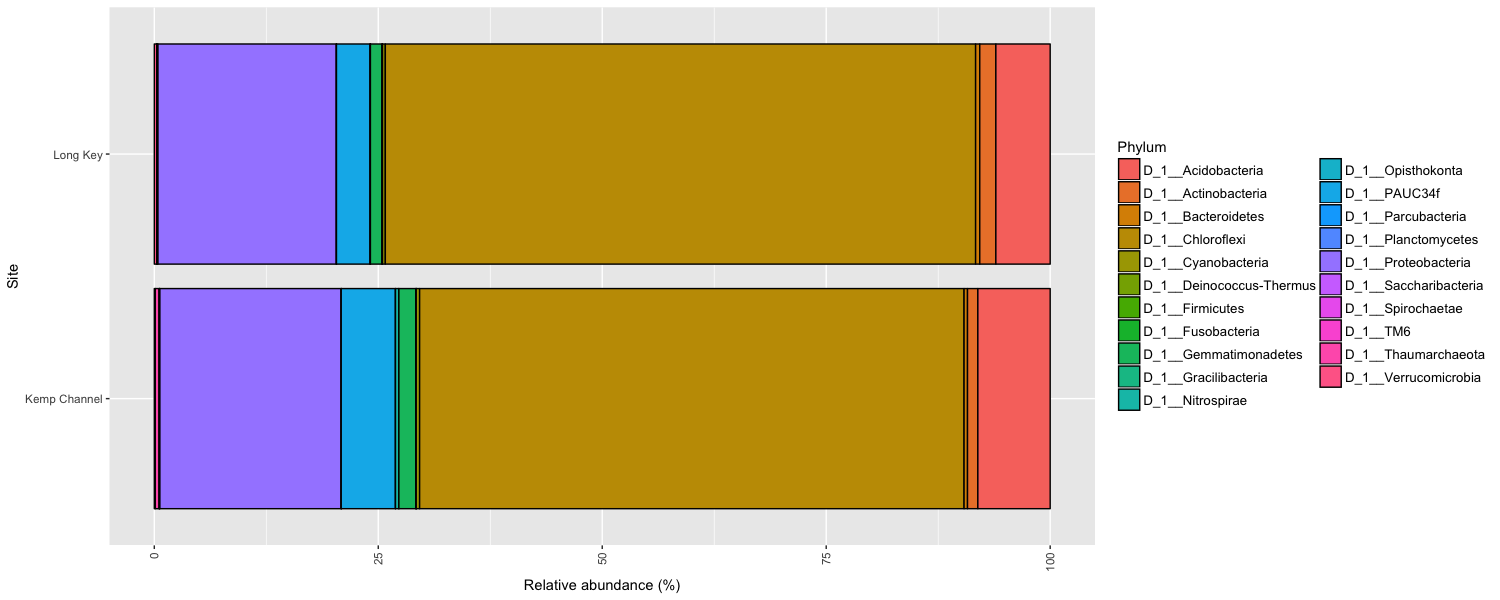
**

**Figure S2**

Relative abundance of microbial phyla identified in *Ircinia campana* from Long Key and Kemp Channel.


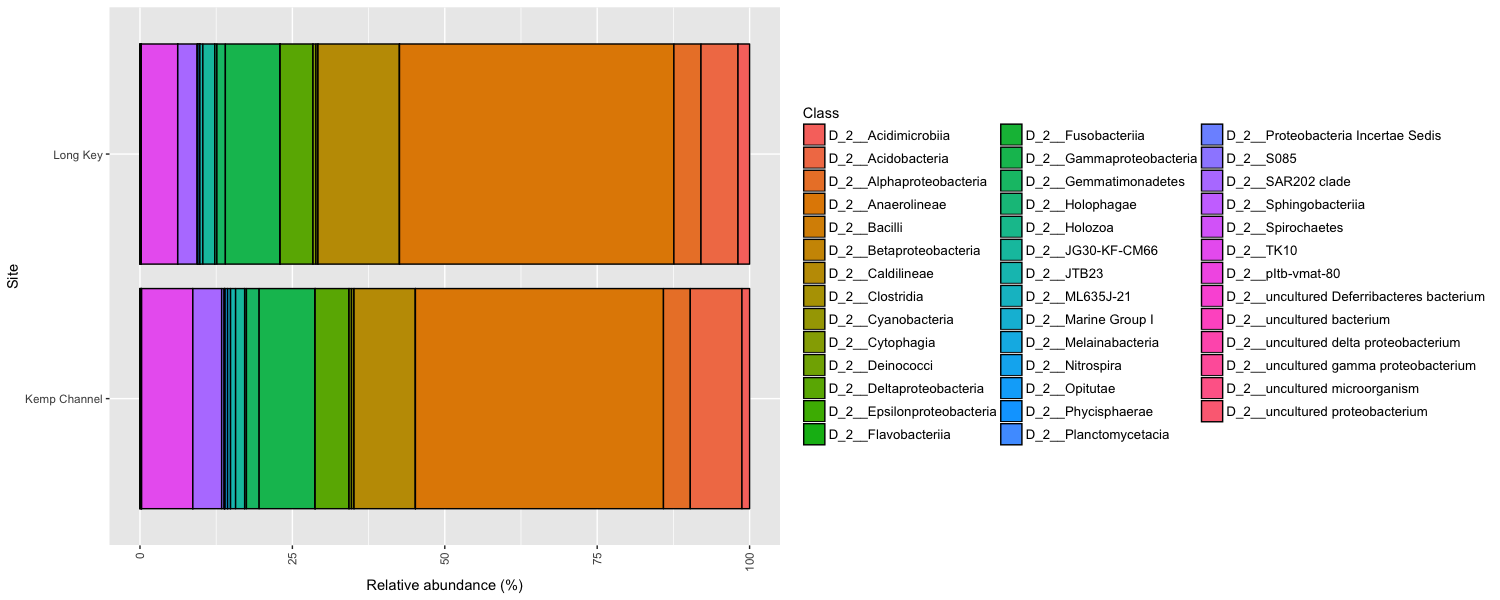


**Figure S3**

Relative abundance of microbial classes identified in *Ircinia campana* from Long Key and Kemp Channel.
